# The Perception of Patient Safety Strategies by Primary Health Professionals

**DOI:** 10.3390/ijerph18031063

**Published:** 2021-01-25

**Authors:** Katarzyna Kosiek, Adam Depta, Iwona Staniec, Michel Wensing, Maciej Godycki-Cwirko, Anna Kowalczyk

**Affiliations:** 1 Family Doctors‘ Clinic, Pomorska 96, 91-402 Lodz, Poland; katarzyna.kosiek@uni.lodz.pl; 2 Department of Medical Insurance and Health Care Financing, Medical University of Lodz, Lindleya 6, 90-131 Lodz, Poland; adam.depta@umed.lodz.pl; 3 Department of Management, Lodz University of Technology, Piotrkowska 266, 90-924 Lodz, Poland; 4 Department of General Practice and Health Services Research, Universitätsklinikum Heidelberg, Im Neuenheimer Feld 672, 69120 Heidelberg, Germany; Michel.Wensing@med.uni-heidelberg.de; 5 Centre for Family and Community Medicine, Faculty of Medical Sciences, Medical University of Lodz, Kopcinskiego 20, 90-153 Lodz, Poland; maciej.godycki-cwirko@umed.lodz.pl (M.G.-C.); anna.kowalczyk@umed.lodz.pl (A.K.)

**Keywords:** patient safety, standard of care, primary care

## Abstract

Almost all European citizens rank patient safety as very or fairly important in their country. However, few patient safety initiatives have been undertaken or implemented in Poland. The aim was to identify patient safety strategies perceived as important in Poland and compare them with those identified in an earlier Dutch study. A web-based survey was conducted among primary healthcare providers in Poland. The findings were compared with those obtained from eight other countries. The strategies regarded as most important in Poland included the use of integrated medical records for communication with specialists and others, patient-held medical records, acceptable workload in general practice, and availability of information technology. However, despite being seen as important, these strategies have not been widely implemented in Poland. This is the first study to identify strategies considered by primary care physicians in Poland to be important for improving patient safety. These strategies differed significantly from those indicated in other countries.

## 1. Introduction

The 2005 Council of Europe definition of patient safety is “freedom from accidental injuries during the course of medical care; activities to avoid, prevent, or correct adverse outcomes which may result from the delivery of healthcare” [1]. The European Commission Directorate General Health and Consumer Protection (SANCO) Eurobarometer survey found that 78% of European citizens ranked medical errors as very or fairly important in their country, and 98% felt that having national political support for patient safety was of high importance [2]. In addition, the World Alliance for Patient Safety has urged member states to develop a coherent strategy for improving patient safety [3], resulting in the development of various safety strategies and validated instruments to promote a culture of patient safety [4,5,6,7,8,9] and research on its implementation in various countries [10,11].

In Poland, most activities related to improving the safety of medical procedures have been local initiatives focused primarily on medication errors. No systematic monitoring of sentinel events (any unanticipated events in a healthcare setting resulting in death or serious physical or psychological injury to a patient, not related to the natural course of the patient’s illness), circumstances, near misses (unplanned events that have the potential to cause, but do not actually result in human injury), or preventable events as described by The Joint Commission (https://www.jointcommission.org/resources/patient-safety-topics/sentinel-event/) has been implemented in general practice, nor has any detailed data been acquired and any root cause analysis of incidents been performed [12,13,14,15]. Furthermore, the health service in Poland suffers from lack of public funding and shortages of medical personnel; these have negative effects on access to healthcare services [8] and, potentially, safety. The aim was (1) to identify and map the patient safety strategies perceived as important in Poland, and (2) to compare the views on importance of these strategies in Poland with those of healthcare professionals in a previous Dutch study [16].

## 2. Materials and Methods

A convenient sample of randomly selected consecutive primary care physicians in Poland were surveyed using an electronic questionnaire. Participants were recruited from several conferences and seminars in Poland where the strategies were presented; most respondents were physicians potentially interested in patient safety. Further contacts were recruited through a snowball sampling procedure. In order to obtain the reliability of the answers, context descriptions were provided. Participation in the study was voluntary. The initial survey was developed in the Netherlands and used in eight countries: Austria, Denmark, France, Germany, the Netherlands, New Zealand, Slovenia, and the United Kingdom [16].

The questionnaire was translated and validated for a Polish context by the study team. It covered five categories: facilities in the practice, patient safety management, communication and collaboration, generic conditions for patient safety, and education regarding patient safety, as well as 37 patient safety strategies (items) including incident reporting, medication alerts, patient safety indicators, periodic medication review, training on patient safety, or culture conditions [16]. A full list of strategies is given in Table 1. A professional research and consulting company collected the questionnaires from the Polish respondents.

The responses regarding the presence of a strategy were “no”, “no, but planned”, “yes, <50% present in the country”, and “yes, >50% present in the country”. Responses concerning the importance of the strategies were “no”, certainly not”, “no, probably not”, “partly yes, partly no”, “yes, to some extent”, and “yes, very much”. The respondent could add a comment to each item. Respondents were also asked to list any other strategies that were not mentioned in the questionnaire.

The percentage of respondents indicating the variants “yes, >50% present in the country” and “yes, very much” were calculated.

The results were compared with those obtained in Dutch study using the two-proportion *z*-test. The following statistical hypotheses were formulated: (H0) the proportion from the studied countries is equal to the proportion from Poland, or (H1) that it is not equal. This was a two-tailed test. The null hypothesis was that no difference existed between the two population proportions or, more formally, that the difference was zero [17]. The *z*-value and associated *p*-value were calculated. The null hypothesis (H0) was rejected if *z* ≥ 1.96 or if *z* ≤ −1.96 or *p* < α = 0.05. The statistical analysis was performed using SPSS 20 (Statistical Package for the Social Sciences, International Business Machines Corporation (IBM), New York, USA).

## 3. Results

During March 2019, 1300 questionnaires were sent to Polish respondents, and 251 replies were received. Out of these 251 replies, three were not completed and, hence, were discarded. Therefore, 248 individuals were included in the study. Of these, 53.2% were male, 56.9% were general practitioners (GPs), 41.1% were in internal medicine, 20% were physicians with just general medical training, and 2% were professionals with other specialties (gynecologist, surgeon, or endocrinologist). Eight percent of the respondents were also involved in teaching and research. Most respondents (66.1%) worked in health centers, 21.4% worked in group practice, and 24.6% ran an individual practice. The physicians differed significantly with regard to the number of patients under medical care of a physician, ranging from 0 to 4000 (mean = 1.763; SD = 873) and the size of their place of work, with the patients in their facility ranging from 300 to 16,220 (mean = 5091; SD = 3096). In addition, 49.6% of respondents conducted their medical practice in cities with over 100,000 inhabitants (Table 2).

### 3.1. Facilities in the Practice

Regarding the “very important for patient safety” category, in the Polish study, the percentage of affirmative responses was much lower than in the Dutch study (the population in the Dutch study by Gaal et al. [16] was almost five times smaller (58 from eight different countries compared to 248 respondents in the present study)) for four strategies: “telephone facilities that allow quick access to the practice, particularly for urgent health problems”, “planned checks of safety of equipment, medication, and other facilities in the practice”, “forms for reporting incidents available”, and “computerized decision support regarding medication safety in daily practice. The percentage of affirmative responses for the remaining strategies was similar to that in the Dutch study.

The Polish respondents were much less likely to indicate the top score (yes: >50% present in the country) than in the Dutch study; this response constituted fewer than 10% of all responses. The response was given in 27% of responses for “planned checks of safety of equipment, medication, and other facilities in the practice”, 15.3% for “forms for reporting incidents available”, 13.7% for “computerized medical record system, which is adequately kept”, and 10.9% for “working agreements with pharmacists when problems arise with delivering medication, e.g., alerts, interaction”. This highlights the rudimentary implementation of these strategies in Poland (Table 1).

### 3.2. Patient Safety Management

The Polish and Dutch studies returned similar percentages of responses “very important for patient safety” for the strategies “nationwide or regional incident reporting weeks” and “campaigns to increase patients’ and public awareness of patient safety in general practice”, while significantly higher percentage responses were given by Poland for “surveys and other types of consultations of patients regarding safety incidents”.

The response “>50% present in the country” was less common in the Polish study than the Dutch study for five strategies, but similar results were observed for the other five (Table 2).

### 3.3. Communication and Collaboration

In this category, the response “very important for patient safety” was significantly more common in the Polish survey than the Dutch survey for the strategies “integrated medical records for communication with specialists and others” and “patient-held medical records”.

The response “>50% present in the country” was recorded at similar frequencies in the Polish and Dutch studies for the following strategies: “structured formats for information on referral of patients”, “the pharmacist conducted periodic reviews of the patient’s medications for potential interactions”, and “patient-held medical records”. For the remaining strategies, it was recorded much less frequently in the Polish study than the Dutch study (Table 1).

### 3.4. Generic Conditions for Patient Safety

In Poland, this category was the most important. The Dutch and Polish studies returned similar percentages of “very important for patient safety” for the strategies “understanding of patient safety in health professionals, particularly regarding how it differs from complications of treatment” and “adequate procedures for identifying and managing burnout in health professionals”. However, a significantly higher percentage was noted for “culture and mentality which facilitates learning from incidents” in the Dutch study than the Polish study, while a significantly higher percentage was returned in the Polish study for “workload is perceived as acceptable in general practice” and “availability of information technology in general practice, and skills to use these adequately” than the Dutch study.

The Polish respondents were significantly less likely to indicate “>50% present in the country” for “availability of information technology in general practice, and skills to use these adequately”. However, similar responses were observed between the Polish and the Dutch studies for perception of presence in the country (Table 1).

### 3.5. Education on Patient Safety

Education was seen as the most important factor for improving patient safety in both studies. In this category, the response “very important for patient safety” was observed more frequently in the Dutch study than the Polish study.

Similar frequencies of “>50% present in the country” were observed between the Polish and Dutch studies for the strategies “education on patient safety in the vocational training of practice nurses” and “postgraduate education on patient safety of practice nurses”. However, it was observed much less frequently in the Polish study for the remaining strategies (Table 1).

## 4. Discussion

This study is one of the first in Poland to identify strategies considered to improve patient safety. It compares the perception of strategies needed to improve patient safety in Poland with those identified in an earlier study carried out in a number of other European countries [13,14,15,16]. Many differences appear to exist between Poland and other European countries with regard to the perceived importance of patient safety strategies. Although 14 of the 37 strategies included in the survey were regarded as being similarly important in the Polish and Dutch studies, very few in Poland perceived them as being implemented in daily practice. This is one of key differences with those obtained by Gaal et al. [16].

In Poland, the most important strategies included “the use of integrated medical records for communication with specialists and others” and “patient-held medical records, acceptable workload in general practice and availability of information technology”. However, despite being seen as important, these strategies have not been widely implemented in Poland; similar results have been obtained in previous studies [15,16]. In the present study, the highest indications were given for “generic conditions for patient safety” and the lowest (all less than 5%) for “patient safety management”; these strategies tended to be less frequently implemented. There is little correlation between the intention of a healthcare worker and the subsequent (improvement) behavior [12,16].

The respondents in Poland ranked all educational items similarly to those in the other countries. However, the implementation of this strategy was found to be lower than in other countries. Hence, including further education on patient safety in vocational training and postgraduate programs in Poland would be desirable. Moreover, a patient safety program could be valuable in education for practices (such as root cause analysis), as noted previously [14,15,16].

## 5. Limitations

The response rate for this study was acceptable. However, most of the respondents were practicing GPs (56.9%), which can be seen as a potential bias. Earlier studies [15] found that, although “regular” practicing GPs found patient safety highly relevant, they tended to have a very broad idea about patient safety. The method used requires a random sample of each population group to compare categorical data and a number sample greater than 100. The total sample size was 248, and the population was not truly definable. Their expertise to speak on the subject was uncertain and may not actually be comparable to the Dutch study, with only 58 respondents [16]. The respondents of the survey showed that the implementation of patient safety strategies is low, probably due to the very low number of respondents (19%), which can be seen as a potential bias. Moreover, among specialties, most of the respondents were practicing GPs which could direct their responses on the basis of their daily work and, therefore, can be seen as another potential bias. Furthermore, the survey only obtained general opinions from the respondent group, and it is difficult to extrapolate system-level changes and recommendations for strategies on the basis of such limited qualitative data.

## 6. Conclusions

The two populations did appear to be significantly different with regard to their opinions. Polish respondents more often declared that “availability of information technology in general practice, and skills to use these adequately”, “acceptable workload in general practice”, and “patient-held medical records” play the most important roles in patient safety. More worryingly, our findings suggest that patient safety management strategies are not perceived as being implemented in Poland at all. The findings may be used to focus the attention of healthcare authorities and professionals on specific safety strategies considered essential by professionals in Poland.

Key points are the following:

- The perception of patient safety by GPs varies between present studies.
- For Polish respondents, the availability of information technology and the skills to use it were most important for patient safety.
- The differences between various countries regarding attitudes toward patient safety should be addressed in the international regulations of medical practice.
- The conclusions are realistic, demonstrating a low implementation of safety management strategies in Polish primary care.

## Figures and Tables

**Table 1:** Views on the importance and implementation of patient safety interventions. GP, general practitioner.

No. ^1^	Strategy (Item)	“Very Important for Patient Safety” (%)		*z*-Value	“>50% Present in the Country” (%)		*z*-Value
		Dutch Study	Polish Study		Dutch Study	Polish Study	
**Facilities in the Practice**							
1	Computerized medical record system, which is adequately kept	82.3	69.8	1.91	82.7	13.7	10.68 ***
2	Telephone facilities that allow quick access to the practice, particularly for urgent health problems	70.7	35.1	4.95 ***	82.7	2.4	14.45 ***
3	Planned checks of safety of equipment, medication, and other facilities in the practice	69	48	2.88 **	53.8	27	3.94 ***
4	Access to web-based clinical guidance tools in daily practice	68	61.7	0.89	57.6	7.3	9.22 ***
5	Forms for reporting incidents available	67.9	40.7	3.74 ***	28.3	15.3	2.33 *
6	Working agreements with pharmacists when problems arise with delivering medication, e.g., alerts, interaction	67.3	55.2	1.68	46.2	10.9	6.36 ***
7	Reminders and alerts regarding safety issues, which are integrated in the medical record system	61.5	62.5	−0.14	43.1	7.3	7.06 ***
8	Computerized decision support regarding medication safety in daily practice	60.8	39.5	2.95 **	44	3.6	8.76 ***
9	Computerized decision support regarding test ordering in daily practice	47.1	46.8	0.04	13.7	1.6	4.29 ***
**Patient Safety Management**							
10	Practice-based reporting and analysis of incidents (e.g., significant event audit)	74.5	39.9	4.76 ***	19.2	4.4	3.92 ***
11	Reporting and analysis of incidents in small educational groups (e.g., quality circles)	66	36.7	4.07 ***	7.7	1.6	2.55 *
12	Measurement and feedback on safety culture in general practices	60.4	28.2	4.65 ***	3.8	0.8	1.77
13	Nationwide or regional educational reporting system for incidents	57.7	36.3	2.99 **	11.5	0.8	4.43 ***
14	Measurement and feedback on indicators for patient safety	57.7	23.4	5.14 ***	5.7	1.6	1.85
15	Hygiene protocols and guidelines present	56.9	33.9	3.24 **	39.6	0.8	9.72 ***
16	Campaigns to increase patients’ and public awareness of patient safety in general practice	39.6	29	1.57	3.8	2.8	0.40
17	Periodic audits by an external inspection authority	38.5	20.6	2.87 **	13.5	5.2	2.26 *
18	Nationwide or regional incident reporting weeks	33.3	31	0.34	2	1.2	0.48
19	Surveys and other types of consultations of patients regarding safety incidents	0	23.8	−4.14 ***	3.8	0.8	1.77
**Communication and Collaboration**							
20	Standards for record keeping (coding, electronic records)	75	64.5	1.53	62.3	14.9	7.62 ***
21	Integrated medical records for communication with specialists and others	65.4	79.4	−2.27 *	9.4	2.8	2.30 *
22	Structured formats for information on referral of patients	61.5	64.5	−0.43	22.6	12.5	1.95
23	Electronic prescriptions and integrated medication overview in the records from the pharmacist	59.6	65.3	−0.82	17.2	1.6	5.13 ***
24	The pharmacist conducted periodic reviews of the patient’s medications for potential interactions	51.9	42.3	1.33	3.8	4	−0.07
25	Comprehensive analysis of prescribing decisions in the pharmacy, using decision support systems	49.1	42.3	0.94	53.8	1.6	11.22 ***
26	Patient-held medical records	41.2	80.6	−6.09 ***	13.2	25	−1.93
**Generic Conditions for Patient Safety**							
27	Culture and mentality which facilitates learning from incidents	73.6	53.2	2.83 **	9.6	14.9	−1.05
28	Understanding of patient safety in health professionals, particularly regarding how it differs from complications of treatment	64.2	56.5	1.07	9.6	21	−2.00
29	Workload is perceived as acceptable in general practice	52.9	71.8	−2.78 **	13.5	10.1	0.75
30	Adequate procedures for identifying and managing burnout in health professionals	50.9	60.9	−1.39	0*	3.6	−1.47
31	Availability of information technology in general practice, and skills to use these adequately	01	75.4	−10.60 ***	34.6	13.3	3.86 ***
	**Education on Patient Safety**						
32	Education on patient safety in the vocational training of GPs	81.1	62.5	2.69 *	23.5	9.3	3.00 **
33	A guideline on patient safety is available	80.9	64.9	2.35 *	15.2	5.2	2.67 *
34	Education on patient safety in the vocational training of practice nurses	79.2	63.3	2.31 *	8.9	7.3	0.41
35	Postgraduate education on patient safety of GPs	78.7	50.4	3.91 ***	13.7	3.6	3.03 **
36	Postgraduate education on patient safety of practice nurses	77.1	59.3	2.52 *	7	4.4	0.83
37	Education on patient safety in the medical curriculum, before graduation	73.6	56	2.46 *	17.3	4	3.69 ***

^1^ consecutive number; * *p* < 0.05, ** *p* < 0.01, *** *p* < 0.001.

**Table 2:** Demographic characteristics in Polish study.

Characteristics	*n*	%
Sex		
Male	132	53.2
Female	116	46.8
Current professional discipline *		
General practitioner	141	56.9
General internist	102	41.1
Other primary care physician	51	20.6
Medical teacher	10	4
Researcher	10	4
Other or unknown discipline (gynecologist, surgeon, or endocrinologist)	6	2.4
Practice		
Individual	61	24.6
Group	53	21.4
Health centers	164	66.1
Number of patients/doctor (value from 0 to 4000), mean and SD	1763	872.96
Number of patients/facility (value from 300 to 16,220), mean and SD	5091	3095.5
Area of practice		
City with over 100,000 inhabitants	123	49.6
City of 30,000 to 100,000 inhabitants	37	14.9
City with less than 30,000 inhabitants	44	17.7
Small town/village	44	17.7

* Some respondents were specialists in more than one discipline.

## Data Availability

The dataset supporting the conclusions of this article is included within the article. A copy of the questionnaire can be obtained from the first authors.

## Abbreviations

CEE	Central and Eastern European
Directorate General’s SANCO	Directorate-General Health and Consumer Protection. This “Directorate-General”was responsible for EU legislation in the areas of food safety, safety of products, public health and consumer safety
GPs	General practitioners
LINNEAUS PC	Learning from International Networks about Errors and Understanding Safety in Primary Care
SPSS 20	Statistical Package for the Social Sciences, version 20
